# The development and validation of a one-off scale to measure procrastination and precrastination traits in young adults

**DOI:** 10.1186/s40359-025-03072-6

**Published:** 2025-07-10

**Authors:** Waqar Husain, Khaled Trabelsi, Achraf Ammar, Zahra Saif, Haitham Jahrami

**Affiliations:** 1 Department of Humanities, COMSATS University Islamabad, Park Road, Islamabad Campus, Islamabad, Pakistan; 2 Research Laboratory Education, Sport Et Santé, High Institute of Sport and Physical Education of Sfax, University of Sfax, EM2S, LR19JS01 Motricité, Tunisia; 3 Department of Movement Sciences and Sports Training, School of Sport Science, The University of Jordan, Amman, Jordan; 4 Department of Training and Movement Science, Institute of Sport Science, Johannes Gutenberg-University Mainz, 55099 Mainz, Germany; 5 Research Laboratory, Faculty of Medicine of Sfax, Molecular Bases of Human Pathology, University of Sfax, LR19ES13 Sfax3000, , Tunisia; 6 Government Hospitals, Manama, Bahrain; 7 Department of Psychiatry, College of Medicine and Health Sciences, Arabian Gulf University, Manama, Bahrain

**Keywords:** Procrastination, Precrastination, Scale Development, Factor Analysis, Reliability, Validity, Cross-Cultural Analysis

## Abstract

**Background:**

Procrastination is the voluntary delay of urgent tasks. Precrastination, on the other hand, is the tendency to complete tasks as soon as possible. The extreme of both these conditions is considered harmful to mental health. There was a significant gap in the measurement of these conditions, and no single tool was discovered to measure both these conditions simultaneously. The current study was the first-ever in this regard, intended to assess a person's inclination toward procrastination or precrastination at the same time.

**Objective:**

The present study aimed to develop and validate a comprehensive scale to measure both procrastination and precrastination traits single-handedly.

**Methods:**

The development of the Procrastination and Precrastination Traits Scale (PPTS) involved generating potential items through literature review, expert feedback, pilot testing, exploratory factor analysis (EFA), and confirmatory factor analysis (CFA). Moreover, the convergent and divergent validity were also evaluated. Data were collected using crowd-sourcing from 5000 participants (women = 60%; aged 18 to 38 years with a mean age of 28 years, SD = 5) from Africa, Asia, Australia, Europe, North America, and South America.

**Results:**

The EFA identified two distinct factors representing procrastination and precrastination, leading to an 18-item scale. The CFA confirmed a good model fit for the two-factor structure of the 18 items. The reliability of both procrastination (ω = 0.86, α = 0.87) and precrastination (ω = 0.79, α = 0.77) was highly satisfying. The fit indices of the CFA reflected strong validity (CFI = 0.94, TLI = 0.93, NNFI = 0.93, and RMSEA = 0.05). The convergent validity of the PPTS was established through the significant positive correlation of its procrastination scale with the Pure Procrastination Scale (*r* = 0.80; p < 0.001). The divergent validity of the PPTS was established through the significant inverse correlation of its procrastination scale with the Satisfaction with Life Scale (*r* = -0.47; *p* < 0.001).

**Conclusions:**

The PPTS is a reliable and valid tool for measuring procrastination and precrastination. The process of developing and validating the PPTS involved data collection from six diverse continents, enabling the PPTS's potential universality and significance.

**Supplementary Information:**

The online version contains supplementary material available at 10.1186/s40359-025-03072-6.

## Introduction

Procrastination, defined as the voluntary delay of urgent tasks, is a prevalent phenomenon with various risk factors and consequences [1, 2]. It is the intentional and irrational act of delaying decisions and choices, even when aware that this delay would result in negative consequences [3, 4]. Procrastination is often understood by researchers as a situation where there is a difference between a person's desire to do something and their actual performance of that activity [5]. Researchers have identified three types of chronic procrastination: avoidant procrastination, which involves avoiding tasks that challenge one's abilities and undermine one's confidence; decisional procrastination, which refers to delaying the process of making decisions; and arousal procrastination, where individuals tend to rush through tasks when faced with a deadline [6]. Academic, decisional, neurotic, and compulsive procrastination depend on cognitive, emotional, and behavioral factors. Decisional procrastination delays decisions, while behavioral procrastination delays actions. Context also distinguishes procrastination [7]. Factors such as personality traits like self-control and conscientiousness, cultural differences, gender disparities, and educational levels have been identified as predictors of procrastination [1, 2].

Procrastination is associated with negative outcomes such as stress, health issues, academic under-achievement, and lower levels of well-being [8, 9]. Moreover, procrastination has been linked to poor sleep habits, including greater social jetlag, shorter sleep duration, insomnia symptoms, and excessive daytime sleepiness, particularly in adolescents and young adults [8, 10].

Psychological factors like evaluation anxiety, task aversiveness, low self-efficacy, fear of failure, and perfectionism are closely associated with procrastination tendencies, leading to lower levels of health, wealth, and well-being [11–13]. Moreover, factors such as lack of motivation, overconfidence, self-esteem issues, and social problems have been identified as contributors to academic procrastination among university students, impacting their learning outcomes and mental well-being [14]. Research has consistently shown that male students tend to procrastinate more than their female counterparts when examining the relationship between gender and procrastination [15–17]. Unmarried individuals procrastinate more than married ones [9, 18, 19]. Studies have also shown positive connections between procrastination, joblessness, unemployment, and poor socioeconomic conditions [16, 20].

Academic procrastination is the last-minute postponing of school responsibilities such as term papers and other academic obligations. Academic postponements are"maladaptive and adaptive forms of delay, and adaptive forms of delay may be more consistent with certain facets of self-regulating learning"[21]. In the context of e-learning, procrastination negatively impacts academic achievement, leading to disengaged learning, increased dropout rates, and poor performance, emphasizing the importance of addressing procrastination in educational settings [22]. Task-related factors such as discomfort, unfamiliarity with tasks, perceived risk (monetary, psychological, social, and physical), and fear of failure can significantly influence procrastination behaviors among students [23].

Procrastination has resulted in significant losses across several domains, including financial, social, and psychological [24]. It has been linked to various negative outcomes, including poor academic and job performance, increased stress and anxiety, and lower well-being [24, 25]. On the other hand, precrastination is a relatively new construct, and its implications for individual and organizational functioning are still being explored [26–28].

The study of procrastination has a rich history, with researchers examining its cognitive, affective, and behavioral underpinnings [29–31]. Procrastination has become a pressing problem in finance and healthcare, as people delay paying their bills and visiting their primary care doctors [32]. It has been extensively studied in psychology and recognized as a pervasive human behavior with significant consequences for well-being and productivity [9, 24, 32]. However, recent research has identified a phenomenon called"precrastination,"which refers to the tendency to complete tasks as soon as possible, even at the expense of extra effort [26–28, 33, 34]. While procrastination and precrastination may seem like opposing tendencies, emerging evidence suggests that they can coexist within the same individual, leading to a complex interplay of motivations and behaviors [26–28, 33, 34]. While procrastination has been extensively studied, the development and manifestation of precrastination tendencies have received less attention. Existing research suggests that precrastination may stem from a desire for closure, a preference for autonomy, or a desire to minimize cognitive load [26–28, 33, 34]. However, the developmental trajectories and potential interactions between procrastination and precrastination remain largely unexplored.

Previous psychometric research has predominantly focused on developing scales to measure procrastination tendencies. Previous studies have yielded the development of self-report questionnaires such as the Pure Procrastination Scale (PPS) [24], the Irrational Procrastination Scale (IPS) [35], the General Procrastination Scale (GPS) [36], the Adult Inventory of Procrastination (AIP) [37], the Tuckman Procrastination Scale (TPS) [38], and the Decisional Procrastination Scale (DPS) [39]. These tools focus on assessing the irrational delay of intended behavior, a core aspect of procrastination.

A growing body of evidence suggests that procrastination and precrastination can coexist within the same individual [26, 27, 30]. This coexistence raises intriguing questions about the underlying mechanisms that govern the expression of these seemingly contradictory tendencies and their potential impacts on individual functioning. Understanding the development and interplay of procrastination and precrastination is crucial for several reasons. First, it can shed light on the cognitive and motivational processes that shape task engagement and time management behaviors, which have significant implications for productivity, well-being, and goal attainment. Second, it can inform the development of targeted interventions and strategies to effectively manage these tendencies, potentially enhancing individual and organizational performance. Third, it can contribute to a more nuanced understanding of individual differences in task engagement and decision-making, which has implications for personnel selection, job design, and training.

While procrastination (delaying tasks) and precrastination (completing tasks prematurely) may appear as opposing tendencies, emerging evidence suggests they represent complementary temporal regulation strategies that can coexist within individuals across different contexts [27]. This dual-process perspective motivated our development of a unified scale, as fragmented measurement risks overlooking critical behavioral patterns—such as how precrastination on routine tasks may enable procrastination on complex ones [40, 41]. By capturing both constructs simultaneously, the PPTS offers researchers a more nuanced tool to examine how these strategies interact dynamically in real-world settings, rather than treating them as mutually exclusive behaviors.

Given the importance of this topic, there is a pressing need for research that examines the development of procrastination and precrastination within the same individuals over time. By studying these tendencies together, researchers can gain insights into their potential interactions, underlying mechanisms, and impacts on various life domains.

The purpose of this study was to develop and validate a psychometric tool designed to measure both procrastination and precrastination traits simultaneously in young adults, thereby addressing a significant gap in existing research, which has typically assessed these behaviors in isolation. Although procrastination (task delay) and precrastination (premature task completion) are increasingly recognized as distinct yet interrelated constructs, a unified scale to evaluate their co-occurrence and differential impacts has not been established. The specific objectives of this study were: Firstly, to create a comprehensive scale that integrates validated indicators of procrastination with emerging markers of precrastination; and secondly, to establish the psychometric properties of this newly developed scale.

The development of a unified scale measuring both procrastination and precrastination stems from critical theoretical and methodological considerations. Existing research has typically examined these behavioral tendencies as discrete constructs, using separate measurement tools that fragment our understanding of individual differences in task completion behaviors. By creating a single integrated scale, we aim to capture the nuanced interplay between procrastination and precrastination within the same individual, recognizing that these are not mutually exclusive but potentially complementary behavioral strategies. This approach allows for a more holistic assessment of an individual's temporal self-regulation, moving beyond binary categorizations to understand the complex cognitive and motivational processes underlying task management. A unified instrument provides researchers and practitioners with a more comprehensive and parsimonious tool for assessing individual differences in task completion behaviors, potentially revealing subtle interactions and trade-offs between premature and delayed task engagement that may be obscured when using separate measurement approaches.

The focus on young adults in this study is both intentional and significant. Young adulthood is a critical developmental stage marked by substantial transitions, heightened personal autonomy, and the emergence of self-regulatory behaviovrs [42]. This life phase is particularly conducive to examining procrastination and precrastination, as individuals concurrently develop academic, professional, and personal time management strategies [43]. During this time, young adults encounter complex task environments in educational settings, early career stages, and personal life domains, making them an ideal population for investigating the emergence and interaction of these contrasting behavioral tendencies [42]. Young adults are uniquely positioned to offer insights into the cognitive and motivational processes that underpin task completion behaviors, as they navigate increasingly sophisticated personal and professional challenges that require refined self-regulation skills.

## Methods

### The development of the scale

To develop the procrastination and precrastination traits scale, we began by generating an extensive list of potential items through a comprehensive review of the literature on procrastination and precrastination. We examined existing scales, such as the pure procrastination scale, the general procrastination scale, the Tuckman procrastination scale, and empirical studies on precrastination. This review helped us identify common behaviors and themes associated with both constructs. From this, we drafted items that encapsulated key aspects of delaying and hastening behaviors, ensuring that each item was clear, concise, and measured a single construct. Once we had a preliminary list of items, we conducted several rounds of refinement. Initial drafts were reviewed for face validity and clarity by a panel of experts in psychology and behavioral research. Feedback from this panel led to the rewording of some items to enhance clarity and reduce ambiguity. We also ensured all items fit a shared response format, asking participants to rate the frequency of each behavior on a 5-point Likert scale ranging from 1 (Never) to 5 (Always) for ease of administration. The preliminary item pool, consisting of 20 items (10 for procrastination and 10 for precrastination), was pilot tested with a small, diverse sample (*N* = 20). Participants provided feedback on the clarity and relevance of each item. We used this feedback to further refine the items, ensuring that they were understandable and accurately captured the intended behaviors.

### Participants

The total sample size included in the final analyses was 5000 participants split into two studies. Study 1 included 259 individuals with no missing values for sex or age. The ages ranged from 18 to 38 years old, with a mean of 28 and a median of 29. The standard deviation of age is 5 years. More of the sample (154, 59%) is female, while fewer (105, 41%) are male. Study 2 included 4,741 individuals with complete information on sex and age. The age range is from 18 to 38 years old, with a mean of 28 and a median of 28. The standard deviation for age is 6 years. The sample is split between females (2,846, 60%) and males (1,859 or 40%). For cross-cultural comparison purposes, participants were reclassified according to their country of origin (not residence) into continental groupings based on the United Nations geoscheme classification system (e.g., participants originally from Tunisia is classified in Africa and Pakistan is classified in Asia).

### Design and procedure

This study employed a cross-sectional survey design to develop and validate the Procrastination and Precrastination Traits Scale. The design consisted of two primary phases: scale development and scale validation. In the first phase, initial items were generated based on theoretical frameworks and existing literature on procrastination and precrastination. Expert reviews and cognitive interviews were conducted to refine the items. In the second phase, the psychometric properties of the scale were evaluated using exploratory and confirmatory factor analyses, along with assessments of reliability and validity. Data was collected online using social media crowd-sourcing platforms.

### Measures

#### The procrastination and precrastination traits scale (PPTS)

The PPTS a psychometric tool that assesses individuals'tendencies towards procrastination and precrastination using 18 items. Originally consisting of 20 items, the scale was refined by removing two items to achieve its final form. Respondents are instructed to rate each statement on a 5-point Likert scale ranging from 1 (Never) to 5 (Always). The scale is divided into two sections. The first section comprises 10 items that evaluate procrastination behaviors, such as delaying important tasks, putting off unpleasant chores, and waiting until the last minute to prepare for deadlines. Sample statements include"I delay getting started on important tasks, even when I know I shouldn't"and"I wait until the last minute to prepare for tests or deadlines."The remaining 8 items measure precrastination tendencies, which involve a prompt and proactive approach to tasks. Examples of these behaviors include completing chores immediately and maintaining organized spaces. Illustrative statements in this section include"I complete tasks as soon as possible to get them out of the way"and"I keep my work and living spaces consistently neat and organized."

#### The pure procrastination scale (PPS)

The Pure Procrastination Scale (PPS) [44] measures procrastination behaviors in various domains of life [44]. The scale consists of 12 items that are rated on a 5-point Likert scale ranging from"1"(very rarely or does not represent me) to"5"(very often or always represents me). The items cover a range of procrastination behaviors such as delay in starting or completing tasks, postponing decisions, and the inability to meet deadlines. The PPS has demonstrated strong psychometric properties in various further studies Cronbach α > 0.75 [19, 44–47].

#### The satisfaction with life scale (SWLS)

The SWL [48]was employed to determine the convergent validity of the PPTS. The SWLS is a well-known and widely used measure for assessing overall cognitive evaluations of life satisfaction. It employs a 7-item Likert scale for responses, with options ranging from 1 (indicating"strongly disagree") to 7 (indicating"strongly agree"). Higher scores indicate higher levels of life satisfaction [48]. The scale's reliability and validity have been demonstrated in numerous studies conducted worldwide [48–51].

### Ethical issues

Ethical approval was granted by the departmental review committee at COMSATS University with the code CUI-ISB/HUM/ERC-CPA/2024–011. All the procedures performed in this study were in accordance with the 1964 Helsinki Declaration and its subsequent amendments.

### Data analysis plan

Before conducting the analyses, the data were visualized to check for normality and identify potential outliers. Appropriate data screening and cleaning procedures were followed to ensure that the assumptions of the statistical tests were met. Several strategies were employed to ensure data integrity. Initially, missing values and outliers were identified through visual inspection of distributions and descriptive statistics, including extreme z-scores (± 3.29). To detect potential automated or careless responses, repetitive sequences, such as consecutive identical answers across items, were flagged algorithmically. Additionally, monotonous response patterns, exemplified by straight-lining"1"or"5"across all items, were analyzed using variance thresholds. Response time stamps were also reviewed to identify implausibly rapid completions, defined < 2 s per item). Suspect cases were cross-verified manually, and participants exhibiting these patterns (only *n* = 11) were excluded to minimize noise. Normality assumptions were confirmed via skewness (−0.8 to 0.7) and kurtosis (−1.1 to 1.3) indices, and multicollinearity was assessed using variance inflation factors (VIF < 5). Descriptive statistics were used for categorical and continuous data.

The data analysis followed several steps. First, an exploratory factor analysis was conducted on the initial 20-item procrastination and precrastination traits scale to examine the underlying factor structure. The number of factors retained in the EFA was determined using a combination of statistical and theoretical criteria. First, the Kaiser-Guttman criterion (eigenvalues > 1) was applied. Second, a scree plot was visually inspected. Items with primary factor loadings < 0.40 or cross-loadings > below 0.40 or cross-loadings above 0.30 were identified for removal to enhance clarity. The maximum likelihood extraction method and no rotation were employed. Factor loadings, uniqueness values, and model fit indices were evaluated to identify and remove problematic items. After scale refinement based on the exploratory analysis, a confirmatory factor analysis was performed on the final 18-item scale using the maximum likelihood estimation method. Multiple fit indices (e.g., CFI, TLI, RMSEA, SRMR) were examined to assess the goodness-of-fit of the model. Factor loadings, residual variances, average variance extracted, and the heterotrait-monotrait ratio were calculated and reported.

To comprehensively evaluate the factor structure of the PPTS, we CFA on both a hypothesized two-factor model and a single-factor model. This approach allowed us to systematically compare the model fit and determine the most appropriate factor structure for the scale.

To evaluate the reliability of the scale, coefficient ω and coefficient α were computed for each factor (procrastination and precrastination). Convergent and divergent validity were assessed by correlating the two factors with established measures of pure procrastination and life satisfaction. Potential differences in procrastination and precrastination scores across geographical regions were investigated using a one-way ANOVA. The assumption of homogeneity of variances was tested using Levene's test. Since the assumption was met and the ANOVA was not significant, post-hoc tests were not conducted. The p-value was set at < 0.05 for all analyses. Commonly accepted thresholds for model fit indices include CFI and TLI ≥ 0.90 (acceptable) and ≥ 0.95 (excellent), RMSEA ≤ 0.08 (acceptable) and ≤ 0.05 (excellent), and SRMR < 0.08 (good fit). Sampling adequacy is guided by KMO ≥ 0.60 (adequate) and ≥ 0.80 (meritorious), with Bartlett’s test (*p* < 0.05) confirming factorability. For validity, AVE ≥ 0.50 supports convergent validity, while HTMT ratios < 0.85–0.90 indicate discriminant validity. Reliability standards suggest ω and α ≥ 0.70 (acceptable) and ≥ 0.80 (good). All analyses were performed using STATA SE 17 software.

## Results

An exploratory factor analysis was conducted on the initial 20-item Procrastination and Precrastination Traits Scale using maximum likelihood extraction and no rotation (*N* = 259). The analysis revealed two distinct factors (based on eigenvalues (> 1), and scree plot inflection), as shown in Table 1. Factor 1 appeared to represent the procrastination construct, with items Proc1 through Proc10 loading highly on this factor (factor loadings ranging from 0.45 to 0.75). These items captured various procrastination tendencies, including delaying important tasks (Proc1), postponing unpleasant obligations (Proc2, Proc3), last-minute preparation (Proc4), self-handicapping beliefs (Proc5), prioritizing distractions over important tasks (Proc6), procrastinating on health habits (Proc7), delaying decision-making (Proc8), leaving communications unanswered (Proc9), and allowing disorganization due to postponement (Proc10). Factor 2 seemed to reflect the precrastination construct, with items Prec1 through Prec3 and Prec5 through Prec9 loading highly (factor loadings ranging from 0.50 to 0.90). These items captured behaviors indicative of precrastination, such as completing tasks immediately (Prec1, Prec2), tackling unpleasant tasks without delay (Prec3), getting ahead of schedule (Prec5), avoiding postponement of decisions (Prec6), prompt communication responses (Prec7), consistent organization (Prec8), and not waiting until deadlines (Prec9). However, two items exhibited problematic factor loadings. Prec4 ("I start working on assignments or projects immediately rather than waiting") had a negative cross-loading of −0.54 on Factor 1, while Prec10 ("At work/school, I complete things well before they are due") also cross-loaded negatively at −0.54 on Factor 1. The uniqueness values for these two items were high (0.75 for both), suggesting they may not be reliable indicators of the intended precrastination construct. Based on these results, Prec4 and Prec10 were identified as potential candidates for removal from the scale. See Table 1.

**Table 1:** Exploratory factor analysis of the initial version of the procrastination and precrastination traits scale (20-items) *N* = 259 participants

Item	Factor Loadings		
	**Factor 1**	**Factor 2**	**Uniqueness**
Proc1	0.70		0.50
Proc2	0.50		0.71
Proc3	0.50		0.64
Proc4	0.45		0.72
Proc5	0.74		0.46
Proc6	0.66		0.59
Proc7	0.63		0.56
Proc8	0.75		0.46
Proc9	0.58		0.68
Proc10	0.47		0.73
Prec1		0.67	0.55
Prec2		0.67	0.59
Prec3		0.50	0.70
Prec4	−0.54		0.75
Prec5		0.50	0.65
Prec6			0.90
Prec7		0.53	0.65
Prec8		0.62	0.61
Prec9		0.62	0.64
Prec10	−0.54		0.75

Maximum likelihood extraction method was used in combination with no rotation

Factor 1 = Procrastination and Factor 2 = Precrastination

Abbreviations ‘Proc’ = Procrastination and ‘Prec’ = Precrastination

Based on exploratory factor analysis items Prec4 and Prec10 were suggested for deletion

Proc1 = I delay getting started on important tasks, even when I know I shouldn't

Proc2 = I keep putting off unpleasant chores or obligations

Proc3 = I find myself aimlessly browsing instead of working

Proc4 = I wait until the last minute to prepare for tests or deadlines

Proc5 = I tell myself I'll get more done"tomorrow"rather than following through today

Proc6 = I waste time on unimportant distractions instead of priorities

Proc7 = I procrastinate on health habits like exercise, diet, or appointments

Proc8 = I unnecessarily delay making even small decisions

Proc9 = I leave emails, messages or calls unanswered for too long

Proc10 = My spaces become cluttered and disorganized from postponing cleaning/organizing

Prec1 = I complete tasks as soon as possible to get them out of the way

Prec2 = I take care of chores or obligations immediately rather than delaying

Prec3 = I tackle unpleasant tasks right away without postponing

Prec4 = I start working on assignments or projects immediately rather than waiting

Prec5 = I try to get things done ahead of schedule when possible

Prec6 = I don't postpone making decisions, even on small matters

Prec7 = I reply to messages, emails, or calls promptly without delay

Prec8 = I keep my work and living spaces consistently neat and organized

Prec9 = I don't wait for deadlines to start on important tasks or goals

Prec10 = At work/school, I complete things well before they are due

A confirmatory factor analysis was conducted on the final 18-item Procrastination and Precrastination Traits Scale with a large sample (*N* = 4741). The analysis revealed a two-factor structure, aligning with the proposed constructs of procrastination (Factor 1) and precrastination (Factor 2). All items loaded significantly on their respective factors, with factor loadings ranging from 0.55 to 0.72 for procrastination items and 0.24 to 0.68 for precrastination items. However, item Prec6 ("I don't postpone making decisions, even on small matters") had a relatively low factor loading of 0.24 on the precrastination factor. The model demonstrated good fit based on multiple indices, including the CFI (0.94), TLI (0.93), NNFI (0.93), and RMSEA (0.05). The SRMR value of 0.04 also indicated good fit. Preliminary analyses supported the use of factor analysis, with a KMO of 0.93 and a significant Bartlett's test. The average variance extracted was 0.39 for the procrastination factor and 0.32 for the precrastination factor. The heterotrait-monotrait ratio of 0.67 suggested some overlap between the two factors. Reliability analyses showed good internal consistency for the procrastination factor (ω = 0.86, α = 0.87) and acceptable reliability for the precrastination factor (ω = 0.79, α = 0.77). See Table 2.

**Table 2:** Confirmatory factor analysis and reliability analysis of the final version of the procrastination and precrastination traits scale (18-items) N = 4741 participants

Factor loadings						Residual variances			
**Factor**	**Indicator**	**Value**	**SE**	**z-value**	**p-value**	**Value**	**SE**	**z-value**	**p-value**
Factor 1	Proc1	0.63	0.02	45.38	< 0.001	0.61	0.02	44.02	< 0.001
	Proc2	0.55	0.02	38.71	< 0.001	0.7	0.03	45.69	< 0.001
	Proc3	0.62	0.02	44.39	< 0.001	0.62	0.03	44.14	< 0.001
	Proc4	0.57	0.02	40.32	< 0.001	0.67	0.03	45.21	< 0.001
	Proc5	0.65	0.02	47.57	< 0.001	0.58	0.02	43.59	< 0.001
	Proc6	0.69	0.02	50.98	< 0.001	0.53	0.02	42.24	< 0.001
	Proc7	0.6	0.02	43.25	< 0.001	0.64	0.03	44.7	< 0.001
	Proc8	0.72	0.02	54.22	< 0.001	0.48	0.02	41.08	< 0.001
	Proc9	0.67	0.02	49.09	< 0.001	0.56	0.02	43.1	< 0.001
	Proc10	0.58	0.02	40.82	< 0.001	0.67	0.03	45.27	< 0.001
Factor 2	Prec1	0.67	0.02	47.71	< 0.001	0.55	0.03	39.84	< 0.001
	Prec2	0.63	0.02	43.64	< 0.001	0.61	0.03	41.72	< 0.001
	Prec3	0.68	0.02	48.47	< 0.001	0.54	0.02	39.45	< 0.001
	Prec5	0.52	0.02	35.35	< 0.001	0.72	0.03	44.64	< 0.001
	Prec6	0.24	0.02	15.36	< 0.001	0.94	0.04	48	< 0.001
	Prec7	0.52	0.02	34.88	< 0.001	0.73	0.03	44.79	< 0.001
	Prec8	0.61	0.02	41.95	< 0.001	0.63	0.03	42.49	< 0.001
	Prec9	0.57	0.02	39.35	< 0.001	0.67	0.03	43.43	< 0.001

Maximum likelihood extraction method was used in combination with no rotation

Factor 1 = Procrastination and Factor 2 = Precrastination

Abbreviations ‘Proc’ = Procrastination and ‘Prec’ = Precrastination

Based on exploratory factor analysis items Prec4 and Prec10 were deleted

Proc1 = I delay getting started on important tasks, even when I know I shouldn't

Proc2 = I keep putting off unpleasant chores or obligations

Proc3 = I find myself aimlessly browsing instead of working

Proc4 = I wait until the last minute to prepare for tests or deadlines

Proc5 = I tell myself I'll get more done"tomorrow"rather than following through today

Proc6 = I waste time on unimportant distractions instead of priorities

Proc7 = I procrastinate on health habits like exercise, diet, or appointments

Proc8 = I unnecessarily delay making even small decisions

Proc9 = I leave emails, messages or calls unanswered for too long

Proc10 = My spaces become cluttered and disorganized from postponing cleaning/organizing

Prec1 = I complete tasks as soon as possible to get them out of the way

Prec2 = I take care of chores or obligations immediately rather than delaying

Prec3 = I tackle unpleasant tasks right away without postponing

^*^Prec4 = I start working on assignments or projects immediately rather than waiting

Prec5 = I try to get things done ahead of schedule when possible

Prec6 = I don't postpone making decisions, even on small matters

Prec7 = I reply to messages, emails, or calls promptly without delay

Prec8 = I keep my work and living spaces consistently neat and organized

Prec9 = I don't wait for deadlines to start on important tasks or goals

^*^Prec10 = At work/school, I complete things well before they are due

^*^Removed from original 20 items version

Model fit

Baseline model = Χ^2^ 25,141.91, df (153), *p* < 0.001

Factor model = Χ^2^ 1677.4, df (153), *p* < 0.001

Fit indices

Comparative Fit Index (CFI) = 0.94

Tucker-Lewis Index (TLI) = 0.93

Bentler-Bonett Non-normed Fit Index (NNFI) = 0.93

Bentler-Bonett Normed Fit Index (NFI) = 0.93

Parsimony Normed Fit Index (PNFI) = 0.82

Bollen's Relative Fit Index (RFI) = 0.92

Bollen's Incremental Fit Index (IFI) = 0.94

Relative Noncentrality Index (RNI) = 0.94

Information criteria

Log-likelihood = −135,407.43

Number of free parameters = 55

Akaike (AIC) = 270,924.85

Bayesian (BIC) = 271,280.37

Sample-size adjusted Bayesian (SSABIC) = 271,105.6

Other fit measures

Root mean square error of approximation (RMSEA) = 0.05

RMSEA 90% CI lower bound = 0.05

RMSEA 90% CI upper bound = 0.05

RMSEA p-value = 0.71

Standardized root mean square residual (SRMR) = 0.04

Hoelter's critical N (α = 0.05) = 458.92

Hoelter's critical N (α = 0.01) = 495.61

Goodness of fit index (GFI) = 0.99

McDonald fit index (MFI) = 0.85

Expected cross validation index (ECVI) = 0.38

Kaiser–Meyer–Olkin (KMO) test = 0.93

Bartlett's test of sphericity = Χ^2^ 25,100.37,, df (153), *p* < 0.001

Average variance extracted

Factor 1 = 0.39

Factor 2 = 0.32

Heterotrait-Monotrait Ratio

Factor 1 to Factor 2 = 0.67

Reliability

For Factor 1, the coefficient ω is 0.86, and the coefficient α is 0.87

For Factor 2, the coefficient ω is 0.79, and the coefficient α is 0.77

When comparing the two-factor and one-factor models, the two-factor model demonstrated substantially better fit indices. The one-factor model exhibited notably lower fit indices, with the CFI at 0.789, TLI at 0.762, and RMSEA at 0.087, which are considerably less optimal compared to the two-factor model's CFI of 0.94 and RMSEA of 0.05. These results suggest that the two-factor structure more accurately represents the underlying construct of procrastination and precrastination traits.

To assess convergent and divergent validity, the final 18-item PPTS was correlated with the established PPS and the SWLS in a large sample (*N* = 4741). The procrastination factor (Factor 1) exhibited a strong positive correlation with the PPS (*r* = 0.80, *p* < 0.001), providing evidence of convergent validity. As expected, the procrastination factor also demonstrated a moderate negative correlation with the SWLS (*r* = −0.47, *p* < 0.001), suggesting that higher procrastination is associated with lower life satisfaction. In contrast, the precrastination factor (Factor 2) had a moderate negative correlation with the PPS (*r* = −0.44, *p* < 0.001) and a weaker positive correlation with the SWLS (*r* = 0.27, *p* < 0.001). These findings align with the conceptualization of precrastination as the opposite of procrastination, with precrastination being negatively related to pure procrastination and positively associated with life satisfaction. The procrastination and precrastination factors also showed a moderate negative correlation with each other (*r* = −0.54, *p* < 0.001), further supporting their divergent nature as contrasting constructs. The results are presented in Table 3.

**Table 3:** Convergent and divergent validity of the final version of the procrastination and precrastination traits scale (18-items) with the 12-item pure procrastination scale (PPS) and the satisfaction with life scale (SWLS) N = 4741 participants

Variables	Factor 1	Factor 2	PPS	SWLS
Factor 1	-			
Factor 2	−0.54*	-		
PPS	0.80*	−0.44*	-	
SWLS	−0.47*	0.27*	−0.39*	-

Factor 1 = Procrastination and Factor 2 = Precrastination of the procrastination and precrastination traits scale (18-items)

PPS = the 12-item Pure Procrastination Scale

SWLS = the satisfaction with life scale

^*^p-value < 0.001

The PPTS was computed for a large sample of 5000 participants from six different continents: Africa (N = 825), Asia (N = 1217), Australia (*N* = 833), Europe (*N* = 885), North America (*N* = 402), and South America (*N* = 838). A one-way ANOVA was conducted to examine differences in procrastination and precrastination scores across the continents.

For procrastination scores, the ANOVA results showed no significant difference between the six continent groups, F(5, 4994) = 1.48, *p* = 0.218. The mean procrastination scores were relatively similar, ranging from 25.77 (North America) to 26.70 (Australia), with small standard deviations between 7.24 (Asia) and 7.90 (North America). Levene's test for homogeneity of variances was not significant (*p* = 0.052), suggesting that the assumption of equal variances was met.

Similarly, for precrastination scores, the one-way ANOVA revealed no significant differences across the six continents, F(5, 4994) = 0.974, *p* = 0.432. The mean precrastination scores ranged from 25.39 (Australia) to 25.91 (Europe), with standard deviations between 6.46 (North America) and 7.04 (Australia). Levene's test for homogeneity of variances was also not significant (*p* = 0.06), indicating homogeneous variances. See Table 4.

**Table 4:** Differences, homogeneity, and variance between the procrastination and precrastination traits scale (18-items) scored between continents N = 5000 participants

Continent	N	Procrastination	Precrastination
Africa	825	26.52 ± 7.46	25.59 ± 6.95
Asia	1217	26.61 ± 7.24	25.45 ± 6.67
Australia	833	26.70 ± 7.29	25.39 ± 7.04
Europe	885	26.46 ± 7.38	25.91 ± 6.97
North America	402	25.77 ± 7.90	25.88 ± 6.46
South America	838	26.05 ± 7.44	25.87 ± 6.89

One-Way ANOVA (Fisher's)

The F-statistic for procrastination is 1.48 with degrees of freedom 5 and 4994. The p-value is 0.218. The F-statistic for precrastination is 0.974 with degrees of freedom 5 and 4994. The p-value is 0.432

Homogeneity of Variances Test (Levene's)

The F-statistic for the homogeneity of variances test for procrastination is 2.2 with degrees of freedom 5 and 4994. The p-value is 0.052. The F-statistic for the homogeneity of variances test for precrastination is 2.17 with degrees of freedom 5 and 4994. The *p*-value is 0.06

## Discussion

We initially developed a novel scale named the PPTS, which contains 20 items. Using a medium sample of 259 participants, the items of the initial version clustered into two distinct factors, aligning with the intended constructs of procrastination and precrastination. However, Prec4 and Prec10 did not load well on either factor, suggesting potential issues with those items. In a larger confirmatory sample, the 18-item scale showed an adequate two-factor structure representing procrastination and precrastination, with most fit indices meeting criteria for good model fit. Reliability was good for the procrastination factor and acceptable for the precrastination factor. There was still some overlap between the two factors based on the heterotrait-monotrait ratio. The correlations observed in this large sample provide evidence for the convergent and divergent validity of the PPTS. The procrastination factor demonstrated strong convergence with an established procrastination measure and showed the expected negative relationship with life satisfaction. Conversely, the precrastination factor exhibited divergence from procrastination tendencies and a positive association with life satisfaction. Our results showed that procrastination and precrastination scores did not significantly differ across the six continents represented in the sample. The variances were relatively homogeneous for both procrastination and precrastination scores. These findings suggest that the PPTS may be measuring constructs that are consistent across diverse geographical regions and cultures.

The items of procrastination developed in our scale refer to the already established indicators of procrastination. Item 1 depicts the delay in initiating tasks (I delay getting started on important tasks, even when I know I shouldn't). Delaying tasks has been regarded as a core aspect of procrastination [52–54]. Item 2 projects the avoidance of unpleasant tasks (I keep putting off unpleasant chores or obligations). The tendency to avoid unpleasant chores or obligations has also been regarded as an integral part of procrastination in earlier studies [55, 56]. Item 3 reflects engagement in distracting activities (I find myself aimlessly browsing instead of working). Spending time on aimless or unimportant activities instead of focusing on work has been regarded as a significant indicator of procrastination [18, 24, 25]. Item 4 depicts last-minute preparation (I wait until the last minute to prepare for tests or deadlines). Studies reveal that preparation for tests or deadlines in procrastination is often delayed until the last minute [57–60]. Item 5 links our scale with rationalization in postponement (I tell myself I'll get more done'tomorrow'rather than following through today). Studies reveal that during procrastination, individuals tend to rationalize their delays and based on their own rationalization, they postpone tasks to the future [60, 61]. Item 6 depicts neglect of priorities (I waste time on unimportant distractions instead of priorities). In procrastination, time is wasted on distractions instead of addressing priorities [27, 62, 63]. Item 7 highlights procrastination on health-related tasks (I procrastinate on health habits like exercise, diet, or appointments). The habits of individuals related to health have also been studied extensively and it has been found that such people delay their health-related appointments [64–66]. Item 8 reflects indecision (I unnecessarily delay making even small decisions). In procrastination, there is unnecessary delay in making even small decisions [13, 19, 29, 67]. Item 9 projects delayed communication (I leave emails, messages, or calls unanswered for too long). Responses to emails, messages, or calls are postponed for too long in procrastination [68]. Item 10 relates to disorganization (My spaces become cluttered and disorganized from postponing cleaning/organizing). Disorganization is regarded as an outcome of procrastination which reflects the disorganized environment such as a bedroom or office where delays in cleanliness can be easily observed [69, 70]. The ten items of our scale that relate to procrastination, therefore, cover both behavioral tendencies and cognitive processes behind behaviors.

The second part of our scale relates to precrastination. In precrastination, people tend to be proactive and desire to complete their tasks immediately. The CFA establishes that 8 out of 10 items of precrastination were statistically validated. Item 1 of our precrastination scale depicts the urgency in completing tasks (I complete tasks as soon as possible to get them out of the way). Previous studies highlight this focus on completing tasks as soon as possible during procrastination [27, 62, 71, 72]. Item 2 reflects the immediate attention to obligations (I take care of chores or obligations immediately rather than delaying). In precrastination, chores or obligations are addressed immediately rather than being delayed [40, 41, 73]. Item 3 highlights the prompt handling of unpleasant tasks (I tackle unpleasant tasks right away without postponing). The tendency to tackle unpleasant tasks right away is a prominent feature of procrastination [56, 74]. Item 5 focuses on the proactive approach to meeting deadlines (I try to get things done ahead of schedule when possible). In precrastination, there is always an effort to complete tasks ahead of schedule whenever possible [27, 33, 40, 41]. Item 6 depicts prompt decision making (I don't postpone making decisions, even on small matters). Literature suggests that people involved in precrastination make their decisions promptly without delay, even on small matters [12, 75, 76]. Item 7 projects timely communication (I reply to messages, emails, or calls promptly without delay). In precrastination, messages, emails, or calls are replied to without delay [21, 40]. Item 8 highlights consistent organization (I keep my work and living spaces consistently neat and organized). A significant tendency of people with precrastination is their ability to keep their work and living spaces neat and organized [24]. The last item of our precrastination scale reflects the proactive initiation of important tasks (I don't wait for deadlines to start on important tasks or goals). It is also prevalent among people with precrastination to start their important tasks or goals well before deadlines [24, 59, 77, 78]. The eight items validated in our precrastination scale, therefore, cover all the known behaviors and attributes of people with precrastination.

The model comparison revealed important insights into the factor structure of procrastination and precrastination. While the one-factor model provided a less satisfactory fit, the two-factor model strongly supported the distinction between procrastination and precrastination as separate but related constructs. This finding aligns with theoretical perspectives suggesting that these traits, while interconnected, represent distinct psychological mechanisms. The lower fit indices of the one-factor model underscore the importance of maintaining the two-factor structure in future research and scale applications.

### Implications

The development and validation of the PPTS have several practical implications for future research and practice. The most significant aspect of our scale is its combination of procrastination and precrastination under one platform. This will enable researchers and clinicians to easily identify if a person is inclined toward procrastination or precrastination. The identification of both of these conditions would further lead to adequate interventions. The PPTS can also be highly beneficial in an organizational context, whereby employers can assess the procrastination and precrastination tendencies of their human resources. Another prominent implication of the PPTS would be in education, whereby teachers and students can use this scale to improve academic achievements.

The development of the PPTS marks a significant advancement in measuring both procrastination and precrastination within a unified framework. However, we recognize several important considerations regarding the interpretation of our findings. First, although the scale exhibits strong psychometric properties, its cross-cultural consistency should be interpreted with caution due to the uneven distribution of our sample across geographic regions. Second, while the observed correlations between the PPTS factors and established measures (*r* = 0.80 with the PPS) provide initial evidence for validity, these relationships primarily validate the procrastination component of our scale. The precrastination dimension necessitates further validation against behavioral measures, as no gold-standard comparison instrument currently exists. Third, the moderate negative correlation between procrastination and life satisfaction (*r* = −0.47), while theoretically significant, should not be overinterpreted, as these constructs likely influence each other through complex, bidirectional pathways. These considerations underscore that our findings, while promising, represent an initial rather than definitive step in understanding how these temporal self-regulation strategies operate independently and in relation to one another.

### Limitations and suggestions

Although we successfully collected online data from 5000 respondents from Africa, Asia, Australia, Europe, North America, and South America, we were unable to include the elderly population.

The primary limitation of this study is its narrow demographic focus, which exclusively sampled young adults. This restricted age range raises significant concerns regarding the generalizability of the PPT) across various life stages and developmental contexts. While our theoretical framework recognizes the complexity of task completion behaviors, the current sample does not adequately represent the potentially distinct manifestations of procrastination and precrastination among older adults, professionals, or individuals in diverse life circumstances.

Additionally, the methodological approach presents further limitations that warrant careful examination. The reliance on self-reported data introduces inherent biases that may undermine the scale's objectivity. Self-report measures are particularly vulnerable to social desirability bias, subjective interpretations by participants, and potential misrepresentations of their own behavioral tendencies. This limitation is especially pronounced when investigating complex psychological constructs such as procrastination and precrastination, where individuals may possess limited self-awareness or motivation to report their behaviors accurately.

Moreover, the cross-sectional design of our study constitutes a significant methodological constraint. Although we successfully established the psychometric properties of the PPTS, the research design precludes any meaningful insights into the developmental trajectories and dynamic interactions of procrastination and precrastination over time. The static nature of our approach fails to capture the potential contextual and temporal variations in task completion behaviors, leaving critical questions about the stability and malleability of these traits unresolved. Finally, we could measure the convergent validity of the Procrastination subscale only and did not assess the convergent validity of the Precrastination subscale, mainly due to the absence of any validated measure on procrastination.

## Conclusion

The current study developed and validated the PPTS involving 500 respondents. The PPTS is unique in that it measures both procrastination and precrastination simultaneously, allowing for a valid differential assessment between these two interconnected mental conditions. We believe that our study represents a significant advancement in the field of procrastination research. The newly developed scale will undoubtedly find applications in educational, organizational, and clinical settings, as well as in social surveys.

## Supplementary Information

Supplementary Material 1.

## Data Availability

Availability of data and materials: The corresponding author can provide the data upon request.

## Abbreviations

AIC: Akaike Information Criterion
ANOVA: Analysis of Variance
CFI: Comparative Fit Index
CFA: Confirmatory Factor Analysis
EFA: Exploratory Factor Analysis
GFI: Goodness of Fit Index
KMO: Kaiser–Meyer–Olkin
MFI: McDonald Fit Index
NNFI: Bentler-Bonett Non-normed Fit Index
PPTS: Procrastination and Precrastination Traits Scale
PPS: Pure Procrastination Scale
RMSEA: Root Mean Square Error of Approximation
SE: Standard Error
SD: Standard Deviation
SRMR: Standardized Root Mean Square Residual
SWLS: Satisfaction with Life Scale
TLI: Tucker-Lewis Index

## References

[1] Alblwi A, et al. Procrastination on social media: predictors of types, triggers and acceptance of countermeasures. Soc Netw Anal Min. 2021;11(1):19.

[2] Villanueva MT. Good things don’t come to those who watchfully wait. Nat Rev Clin Oncol. 2011;8(7):386–386. 21647197 10.1038/nrclinonc.2011.84

[3] Simpson WK, Pychyl TA. In search of the arousal procrastinator: Investigating the relation between procrastination, arousal-based personality traits and beliefs about procrastination motivations. Personality Individ Differ. 2009;47(8):906–11.

[4] Sirois FM. “I’ll look after my health, later”: A replication and extension of the procrastination–health model with community-dwelling adults. Personality Individ Differ. 2007;43(1):15–26.

[5] Blunt A, Pychyl TA. Project systems of procrastinators: A personal project-analytic and action control perspective. Personality Individ Differ. 2005;38(8):1771–80.

[6] Ferrari, J.R., J.L. Johnson, and W.G. McCown, Procrastination and task avoidance: Theory, research, and treatment. 1995: Springer Science & Business Media.

[7] Haghbin, M., Attachment styles and psychological separation in relation to procrastination: a psychodynamic perspective on the breakdown in volitional action. 2006, Carleton University.

[8] Li X, et al. Do procrastinators get worse sleep? Cross-sectional study of US adolescents and young adults. SSM-Population Health. 2019;10: 100518. 31799365 10.1016/j.ssmph.2019.100518 PMC6881694

[9] Steel P, Ferrari J. Sex, education and procrastination: An epidemiological study of procrastinators’ characteristics from a global sample. Eur J Pers. 2013;27(1):51–8.

[10] Johansson F, et al. Associations between procrastination and subsequent health outcomes among university students in Sweden. JAMA Netw Open. 2023;6(1):e2249346–e2249346. 36598789 10.1001/jamanetworkopen.2022.49346 PMC9857662

[11] Ang N. Procrastination as Rational Weakness of Will. J Value Inq. 2012;46(4):403–16.

[12] Ma B, Zhang Y. Precrastination and Time Perspective: Evidence from Intertemporal Decision-Making. Behav Sci. 2023;13(8):631. 37622770 10.3390/bs13080631 PMC10451289

[13] Zhang, P. and W.J. Ma. Procrastination in rational agents. in 2019 Conference on Cognitive Computational Neuroscience. 10.32470/CCN. 2019.

[14] Abbasi, I. and N. Alghamdi, The Prevalence, Predictors, Causes, Treatment, and Implications of Procrastination Behaviors in General, Academic, and Work Setting. International Journal of Psychological Studies, 2015. 7.

[15] Balkis, M. and E. Duru, Prevalence of academic procrastination behavior among pre-service teachers, and its relationship with demographics and individual preferences. Journal of Theory & Practice in Education (JTPE)/Eğitimde Kuram ve Uygulama, 2009. 5(1).

[16] Beutel ME, et al. Procrastination, distress and life satisfaction across the age range–a German representative community study. PLoS ONE. 2016;11(2): e0148054. 26871572 10.1371/journal.pone.0148054 PMC4752450

[17] Klassen RM, et al. A cross-cultural study of adolescent procrastination. J Res Adolesc. 2009;19(4):799–811.

[18] Ferrari JR, Tice DM. Procrastination as a self-handicap for men and women: A task-avoidance strategy in a laboratory setting. J Res Pers. 2000;34(1):73–83.

[19] Svartdal F, et al. On the measurement of procrastination: Comparing two scales in six European countries. Front Psychol. 2016;7:1307. 27630595 10.3389/fpsyg.2016.01307 PMC5005418

[20] Nguyen B, Steel P, Ferrari JR. Procrastination’s impact in the workplace and the workplace’s impact on procrastination. Int J Sel Assess. 2013;21(4):388–99.

[21] Corkin DM, Shirley LY, Lindt SF. Comparing active delay and procrastination from a self-regulated learning perspective. Learn Individ Differ. 2011;21(5):602–6.

[22] You JW. Examining the effect of academic procrastination on achievement using LMS data in e-learning. J Educ Technol Soc. 2015;18(3):64–74.

[23] Ackerman, D.S. and B.L. Gross, The dark side:'uncomfortable'motivational task characteristics affecting procrastination. 2006.

[24] Steel P. The nature of procrastination: a meta-analytic and theoretical review of quintessential self-regulatory failure. Psychol Bull. 2007;133(1):65. 17201571 10.1037/0033-2909.133.1.65

[25] Tice DM, Baumeister RF. Longitudinal study of procrastination, performance, stress, and health: The costs and benefits of dawdling. Psychol Sci. 1997;8(6):454–8.

[26] Rosenbaum DA, Dettling J. Carrying groceries: More items in early trips than in later trips or the reverse? Implications for pre-crastination Psychological Research. 2023;87(2):474–83. 35438320 10.1007/s00426-022-01681-z

[27] Rosenbaum DA, et al. Sooner rather than later: Precrastination rather than procrastination. Curr Dir Psychol Sci. 2019;28(3):229–33.

[28] Rosenbaum DA, Gong L, Potts CA. Pre-crastination: Hastening subgoal completion at the expense of extra physical effort. Psychol Sci. 2014;25(7):1487–96. 24815613 10.1177/0956797614532657

[29] Klingsieck KB. Procrastination: When good things don’t come to those who wait. Eur Psychol. 2013;18(1):24–34.

[30] Shu SB, Gneezy A. Procrastination of enjoyable experiences. J Mark Res. 2010;47(5):933–44.

[31] Sirois F, Pychyl T. Procrastination and the priority of short-term mood regulation: Consequences for future self. Soc Pers Psychol Compass. 2013;7(2):115–27.

[32] Steel P. Arousal, avoidant and decisional procrastinators: Do they exist? Personality Individ Differ. 2010;48(8):926–34.

[33] Rosenbaum DA, Sauerberger KS. Deciding what to do: Observations from a psycho-motor laboratory, including the discovery of pre-crastination. Behav Proc. 2022;199: 104658. 10.1016/j.beproc.2022.104658 35526661

[34] Rosenbaum DA, Sturgill HB, Feghhi I. Think then act, or act then think? Double-response reaction times shed light on decision dynamics in precrastination. In: Cognitive Control of Action. Routledge; 2024. p. 226–57. 10.1037/xge0001253 35708953

[35] Steel, P.D.G., The measurement and nature of procrastination. 2002: University of Minnesota.

[36] Sirois FM, Yang S, van Eerde W. Development and validation of the General Procrastination Scale (GPS-9): A short and reliable measure of trait procrastination. Personality Individ Differ. 2019;146:26–33.

[37] McCown, W. and J. Johnson, The adult inventory of procrastination revised. Unpublished manual, 2001.

[38] Tuckman BW. The development and concurrent validity of the procrastination scale. Educ Psychol Measur. 1991;51(2):473–80.

[39] Mann L. Procrastination revisited: a commentary. Aust Psychol. 2016;51(1):47–51.

[40] Wasserman EA. Precrastination: The fierce urgency of now. Learn Behav. 2019;47:7–28. 30264372 10.3758/s13420-018-0358-6

[41] Wasserman EA, Brzykcy SJ. Pre-crastination in the pigeon. Psychon Bull Rev. 2015;22:1130–4. 25361822 10.3758/s13423-014-0758-3

[42] Scales PC, et al. The dimensions of successful young adult development: A conceptual and measurement framework. Appl Dev Sci. 2015;20(3):150–74. 30344455 10.1080/10888691.2015.1082429 PMC6176765

[43] Limone, P., et al., Examining Procrastination among University Students through the Lens of the Self-Regulated Learning Model. Behavioral Sciences, 2020. 10(12). 10.3390/bs10120184 PMC7760344 33271776

[44] Steel, P., Pure procrastination scale. Personality and Individual Differences, 2010.

[45] Rozental A, et al. Internet-based cognitive-behavior therapy for procrastination: A randomized controlled trial. J Consult Clin Psychol. 2015;83(4):808–24. 25939016 10.1037/ccp0000023

[46] Wijaya H, Tori A. Exploring the Role of Self-Control on Student Procrastination. International Journal of Research in Counseling and Education. 2018;1:13.

[47] Zuber S, et al. Do self-reports of procrastination predict actual behavior? Int J Methods Psychiatr Res. 2020;29(4):1–6. 32530112 10.1002/mpr.1843 PMC7723175

[48] Diener E, et al. The Satisfaction With Life Scale. J Pers Assess. 1985;49(1):71–5. 16367493 10.1207/s15327752jpa4901_13

[49] Diener E, et al. Subjective well-being: Three decades of progress. Psychol Bull. 1999;125(2):276–302.

[50] Review of the Satisfaction With Life Scale. 1993, American Psychological Association: US. p. 164–172.

[51] Pavot W, Diener E. The Satisfaction With Life Scale and the emerging construct of life satisfaction. J Posit Psychol. 2008;3(2):137–52.

[52] Bobe, J., et al., Delaying academic tasks and feeling bad about it: Development and validation of a six-item scale measuring academic procrastination. European Journal of Psychological Assessment, 2022.

[53] Svartdal F, et al. Measuring implemental delay in procrastination: Separating onset and sustained goal striving. Personality Individ Differ. 2020;156: 109762.

[54] Pinxten, M., et al., Purposeful delay and academic achievement. A critical review of the Active Procrastination Scale. Learning and Individual Differences, 2019. 73: p. 42–51.

[55] Van Eerde W. Procrastination: Self-regulation in initiating aversive goals. Appl Psychol. 2000;49(3):372–89.

[56] Milgram NA, Sroloff B, Rosenbaum M. The procrastination of everyday life. J Res Pers. 1988;22(2):197–212.

[57] Loeffler SN, et al. Fostering self-regulation to overcome academic procrastination using interactive ambulatory assessment. Learn Individ Differ. 2019;75: 101760.

[58] Bisin A, Hyndman K. Present-bias, procrastination and deadlines in a field experiment. Games Econom Behav. 2020;119:339–57.

[59] Herweg F, Müller D. Performance of procrastinators: On the value of deadlines. Theor Decis. 2011;70:329–66.

[60] Fischer C. Read this paper later: procrastination with time-consistent preferences. J Econ Behav Organ. 2001;46(3):249–69.

[61] Feng, Y., X. Huang, and S. Liu. Research on academic procrastination among OEC students. in 2018 8th International Conference on Management, Education and Information (MEICI 2018). 2018. Atlantis Press.

[62] Kovoor JG, et al. ’Hurry up’syndrome harms surgical patients and staff. ANZ J Surg. 2023;93(7–8):1756–7. 37458228 10.1111/ans.18593

[63] Häfner A, Oberst V, Stock A. Avoiding procrastination through time management: An experimental intervention study. Educ Stud. 2014;40(3):352–60.

[64] Crutchfield, T.M. and C.E. Kistler, Getting patients in the door: medical appointment reminder preferences. Patient preference and adherence, 2017: p. 141–150. 10.2147/PPA.S117396 PMC5279837 28182131

[65] Lacy NL, et al. Why we don’t come: patient perceptions on no-shows. The Annals of Family Medicine. 2004;2(6):541–5. 15576538 10.1370/afm.123 PMC1466756

[66] Liu N, et al. When waiting to see a doctor is less irritating: Understanding patient preferences and choice behavior in appointment scheduling. Manage Sci. 2018;64(5):1975–96.

[67] Svartdal F, Steel P. Irrational delay revisited: examining five procrastination scales in a global sample. Front Psychol. 2017;8:1927. 29163302 10.3389/fpsyg.2017.01927 PMC5676095

[68] Gafni R, Geri N. Time management: Procrastination tendency in individual and collaborative tasks. Interdiscip J Inf Knowl Manag. 2010;5:115.

[69] Howell AJ, Watson DC. Procrastination: Associations with achievement goal orientation and learning strategies. Personality Individ Differ. 2007;43(1):167–78.

[70] Lay CH. At last, my research article on procrastination. J Res Pers. 1986;20(4):474–95.

[71] Richter M. Commentary: Pre-crastination: hastening subgoal completion at the expense of extra physical effort. Front Psychol. 2015;6:1269. 26379593 10.3389/fpsyg.2015.01269 PMC4546999

[72] Ghafurian M, Reitter D. Impatience in timing decisions: Effects and moderation. Timing & Time Perception. 2018;6(2):183–219.

[73] Zentall TR. Basic behavioral processes involved in procrastination. Front Psychol. 2021;12: 769928. 34887816 10.3389/fpsyg.2021.769928 PMC8649618

[74] Prem, R., et al., Procrastination in Daily Working Life: A Diary Study on Within-Person Processes That Link Work Characteristics to Workplace Procrastination. Frontiers in Psychology, 2018. 9. 10.3389/fpsyg.2018.01087 PMC6042014 30026712

[75] Selim, M. Procrastination in decision making and its possible effects on potential profits. in 2021 International Conference on Decision Aid Sciences and Application (DASA). 2021. IEEE.

[76] KS, V.M., et al., Influence of decision-making styles and affective styles on academic procrastination among students. Cogent Education, 2023. 10(1): p. 2203598.

[77] Ariely D, Wertenbroch K. Procrastination, deadlines, and performance: Self-control by precommitment. Psychol Sci. 2002;13(3):219–24. 12009041 10.1111/1467-9280.00441

[78] Knowles S, et al. Procrastination and the non-monotonic effect of deadlines on task completion. Econ Inq. 2022;60(2):706–20.

