# Supplementary material for: The development and validation of a one-off scale to measure procrastination and precrastination traits in young adults
Source: BMC Psychol. 2025 Jul 10;13:773. doi: 10.1186/s40359-025-03072-6 (PMC12247415; doi:10.1186/s40359-025-03072-6)
Supplement: Supplementary file 1 — Supplementary Material 1. [file 40359_2025_3072_MOESM1_ESM.docx]

**Supplemental material 1: The procrastination and precrastination traits scale (20 and 18-items)**

**Notes: * removed from the initial version. The initial version included 20 items and the final version included 18 items.**

Instructions: For each statement below, rate using the following scale:

1 = Never, 2 = Rarely, 3 = Sometimes, 4 = Often, 5 = Always

**Procrastination Items:**

1. I delay getting started on important tasks, even when I know I shouldn't.

2. I keep putting off unpleasant chores or obligations.

3. I find myself aimlessly browsing instead of working.

4. I wait until the last minute to prepare for tests or deadlines.

5. I tell myself I'll get more done "tomorrow" rather than following through today.

6. I waste time on unimportant distractions instead of priorities.

7. I procrastinate on health habits like exercise, diet, or appointments.

8. I unnecessarily delay making even small decisions.

9. I leave emails, messages or calls unanswered for too long.

10. My spaces become cluttered and disorganized from postponing cleaning/organizing.

**Precrastination Items:**

11. I complete tasks as soon as possible to get them out of the way.

12. I take care of chores or obligations immediately rather than delaying.

13. I tackle unpleasant tasks right away without postponing.

*14. I start working on assignments or projects immediately rather than waiting.

15. I try to get things done ahead of schedule whenever possible.

16. I don't postpone making decisions, even on small matters.

17. I reply to messages, emails, or calls promptly without delay.

18. I keep my work and living spaces consistently neat and organized.

19. I don't wait for deadlines to start on important tasks or goals.

*20. At work/school, I complete things well before they are due.
